# Supplementary material for: A Likelihood Approach for Real-Time Calibration of Stochastic Compartmental Epidemic Models
Source: PLoS Comput Biol. 2017 Jan 17;13(1):e1005257. doi: 10.1371/journal.pcbi.1005257 (PMC5240920; doi:10.1371/journal.pcbi.1005257)
Supplement: S1 File — (TAR.GZ) [file pcbi.1005257.s014.tar.gz › HSPH_Online-SI-Revision/IPoi09/n100-mild/n100-mild_BENCH2_table8.pdf]

1	newly 3 weeks spec	114.	428.929	{144., 802.}	278.091	0.474749	newly 3 weeks cumul	602.	1994.36	{642., 3849.}	233.147	0.423159	newly 5 weeks spec	30.	186.786	{58., 381.}	522.619	0.677872	newly 5 weeks cumul	708.	2466.24	{779., 4778.}	249.617	0.440539	newly 44 weeks cumul	814.	2837.5	{934., 5513.}	249.64	0.439887
2	newly 3 weeks spec	204.	679.808	{233., 1220.}	235.407	0.401006	newly 3 weeks cumul	827.	2965.9	{1020., 5173.}	258.908	0.432639	newly 5 weeks spec	99.	335.942	{108., 570.}	241.317	0.404274	newly 5 weeks cumul	1036.	3790.67	{1308., 6520.}	266.155	0.438788	newly 43 weeks cumul	1187.	4582.29	{1561., 7876.}	286.039	0.459485
3	newly 3 weeks spec	108.	175.976	{83., 277.}	74.8645	0.220915	newly 3 weeks cumul	712.	905.171	{390., 1446.}	50.5275	0.181655	newly 5 weeks spec	44.	70.561	{27., 110.}	75.7761	0.228286	newly 5 weeks cumul	820.	1086.39	{451., 1716.}	53.6704	0.185799	newly 46 weeks cumul	845.	1204.41	{496., 1909.}	61.2614	0.200821
4	newly 3 weeks spec	19.	42.6	{15., 93.}	133.971	0.298218	newly 3 weeks cumul	149.	290.6	{102., 631.}	108.725	0.263636	newly 5 weeks spec	0.1	13.	{3., 32.}	12900.	1.95755	newly 5 weeks cumul	155.	326.945	{110., 721.}	121.935	0.280526	newly 50 weeks cumul	155.	341.473	{121., 744.}	129.689	0.290252
5	newly 3 weeks spec	147.	175.976	{83., 277.}	46.7231	0.177984	newly 3 weeks cumul	785.	905.171	{390., 1446.}	44.7258	0.176581	newly 5 weeks spec	78.	70.561	{27., 110.}	42.4953	0.2161	newly 5 weeks cumul	977.	1086.39	{451., 1716.}	43.0412	0.175864	newly 46 weeks cumul	1144.	1204.41	{496., 1909.}	41.9986	0.182312
6	newly 3 weeks spec	116.	315.083	{111., 661.}	174.749	0.350069	newly 3 weeks cumul	649.	1509.02	{553., 3028.}	138.229	0.300538	newly 5 weeks spec	30.	141.208	{49., 261.}	370.694	0.569573	newly 5 weeks cumul	747.	1861.02	{673., 3702.}	153.143	0.321086	newly 44 weeks cumul	826.	2136.23	{759., 4213.}	162.25	0.333735
7	newly 3 weeks spec	14.	52.6977	{15., 86.}	280.399	0.426592	newly 3 weeks cumul	126.	346.512	{114., 546.}	181.875	0.308115	newly 5 weeks spec	0.1	15.5581	{3., 31.}	15462.8	1.95126	newly 5 weeks cumul	128.	391.326	{129., 615.}	210.193	0.340469	newly 49 weeks cumul	128.	408.953	{134., 649.}	223.637	0.35734
8	newly 3 weeks spec	39.	385.556	{113., 682.}	888.604	0.820316	newly 3 weeks cumul	302.	1731.07	{576., 2907.}	473.201	0.602745	newly 5 weeks spec	15.	182.511	{56., 310.}	1116.74	0.904221	newly 5 weeks cumul	333.	2175.49	{726., 3643.}	553.3	0.655621	newly 43 weeks cumul	361.	2550.13	{858., 4138.}	606.408	0.686888
9	newly 3 weeks spec	85.	248.891	{79., 470.}	197.775	0.320589	newly 3 weeks cumul	398.	1305.55	{432., 2579.}	231.581	0.368822	newly 5 weeks spec	41.	89.2727	{27., 162.}	134.767	0.260536	newly 5 weeks cumul	509.	1545.62	{518., 3017.}	208.309	0.341071	newly 48 weeks cumul	521.	1687.36	{559., 3300.}	227.897	0.365264
10	newly 3 weeks spec	178.	389.532	{132., 853.}	129.476	0.281014	newly 3 weeks cumul	763.	1737.45	{589., 3832.}	137.947	0.29755	newly 5 weeks spec	52.	184.915	{61., 383.}	258.142	0.442153	newly 5 weeks cumul	913.	2186.23	{740., 4810.}	148.615	0.312478	newly 42 weeks cumul	1004.	2604.13	{844., 5643.}	166.809	0.335285
11	newly 3 weeks spec	68.	468.98	{181., 800.}	589.677	0.729958	newly 3 weeks cumul	409.	2215.16	{857., 3763.}	441.603	0.631995	newly 5 weeks spec	9.	206.373	{69., 344.}	2193.03	1.24359	newly 5 weeks cumul	430.	2734.37	{1075., 4685.}	535.901	0.700257	newly 46 weeks cumul	447.	3124.63	{1203., 5282.}	599.022	0.740879
12	newly 3 weeks spec	387.	813.278	{303., 1688.}	119.624	0.271864	newly 3 weeks cumul	1436.	3439.94	{1313., 6930.}	144.234	0.307254	newly 5 weeks spec	224.	418.167	{165., 862.}	102.24	0.250737	newly 5 weeks cumul	1967.	4437.81	{1721., 8957.}	132.177	0.289418	newly 42 weeks cumul	2777.	5482.67	{2080., 11071.}	109.476	0.259059
13	newly 3 weeks spec	44.	172.16	{67., 305.}	292.545	0.522669	newly 3 weeks cumul	425.	906.52	{386., 1554.}	119.539	0.292197	newly 5 weeks spec	11.	64.94	{26., 105.}	490.364	0.693846	newly 5 weeks cumul	440.	1077.48	{461., 1857.}	148.455	0.337027	newly 47 weeks cumul	442.	1182.82	{507., 2038.}	170.439	0.370133
14	newly 3 weeks spec	73.	80.	{22., 127.}	74.6575	0.287683	newly 3 weeks cumul	262.	480.393	{131., 828.}	110.305	0.243676	newly 5 weeks spec	18.	23.7143	{6., 38.}	84.3254	0.281819	newly 5 weeks cumul	346.	550.107	{151., 941.}	94.2197	0.237785	newly 49 weeks cumul	354.	578.536	{162., 1013.}	96.6404	0.237107
15	newly 3 weeks spec	78.	440.818	{137., 913.}	465.152	0.604379	newly 3 weeks cumul	442.	2081.49	{681., 4276.}	370.926	0.535757	newly 5 weeks spec	22.	192.527	{57., 417.}	775.124	0.785564	newly 5 weeks cumul	504.	2563.2	{808., 5233.}	408.571	0.566099	newly 46 weeks cumul	595.	2925.44	{914., 5982.}	391.67	0.550531
16	newly 3 weeks spec	250.	704.22	{256., 1079.}	188.732	0.342418	newly 3 weeks cumul	1232.	2904.76	{1124., 4615.}	144.051	0.300717	newly 5 weeks spec	117.	371.439	{115., 541.}	223.723	0.356218	newly 5 weeks cumul	1525.	3795.2	{1416., 5927.}	156.916	0.311441	newly 42 weeks cumul	1724.	4690.68	{1755., 7110.}	179.302	0.334066
17	newly 3 weeks spec	47.	237.842	{84., 421.}	406.047	0.573478	newly 3 weeks cumul	382.	1256.37	{449., 2098.}	229.508	0.398361	newly 5 weeks spec	36.	87.614	{31., 163.}	148.538	0.286265	newly 5 weeks cumul	445.	1488.12	{540., 2498.}	234.969	0.405218	newly 48 weeks cumul	515.	1628.26	{601., 2736.}	216.869	0.381199
18	newly 3 weeks spec	0.1	20.3962	{8., 36.}	20296.2	2.2208	newly 3 weeks cumul	28.	147.679	{56., 266.}	427.426	0.646963	newly 5 weeks spec	0.1	4.98113	{1., 12.}	4892.45	1.45372	newly 5 weeks cumul	28.	162.547	{67., 303.}	480.526	0.686282	newly 52 weeks cumul	28.	166.792	{73., 315.}	495.687	0.697233
19	newly 3 weeks spec	126.	673.294	{265., 1155.}	434.36	0.63753	newly 3 weeks cumul	683.	2983.45	{1195., 5103.}	336.816	0.553284	newly 5 weeks spec	78.	331.098	{124., 556.}	324.485	0.530504	newly 5 weeks cumul	871.	3788.9	{1524., 6529.}	335.006	0.550082	newly 43 weeks cumul	985.	4545.1	{1805., 7905.}	361.431	0.574057
20	newly 3 weeks spec	0.1	43.2979	{6., 148.}	43197.9	2.39813	newly 3 weeks cumul	49.	322.723	{69., 899.}	559.401	0.616666	newly 5 weeks spec	0.1	10.8511	{1., 35.}	10768.1	1.68383	newly 5 weeks cumul	49.	355.	{77., 998.}	624.663	0.654847	newly 52 weeks cumul	49.	364.34	{77., 1016.}	643.726	0.666696
21	newly 3 weeks spec	25.	20.3962	{8., 36.}	46.1887	0.262379	newly 3 weeks cumul	110.	147.679	{56., 266.}	61.4237	0.209565	newly 5 weeks spec	10.	4.98113	{1., 12.}	68.6792	0.602307	newly 5 weeks cumul	146.	162.547	{67., 303.}	50.4135	0.205159	newly 52 weeks cumul	154.	166.792	{73., 315.}	49.5957	0.208033
22	newly 3 weeks spec	390.	645.827	{271., 1362.}	82.3422	0.228199	newly 3 weeks cumul	1699.	2795.77	{1148., 5810.}	80.5904	0.223931	newly 5 weeks spec	240.	325.731	{143., 668.}	65.2724	0.216422	newly 5 weeks cumul	2286.	3582.52	{1476., 7407.}	75.6637	0.218774	newly 42 weeks cumul	2969.	4380.69	{1827., 9013.}	70.7037	0.21652
23	newly 3 weeks spec	51.	351.654	{122., 528.}	589.517	0.669811	newly 3 weeks cumul	271.	1749.4	{610., 2856.}	545.536	0.656238	newly 5 weeks spec	0.1	143.981	{40., 212.}	143881.	2.97312	newly 5 weeks cumul	288.	2122.44	{718., 3449.}	636.959	0.709805	newly 47 weeks cumul	288.	2371.02	{815., 3879.}	723.271	0.754947
24	newly 3 weeks spec	1.	20.66	{5., 43.}	1966.	1.16896	newly 3 weeks cumul	56.	146.12	{46., 317.}	168.071	0.335242	newly 5 weeks spec	0.1	4.76	{0., 11.}	4692.	1.35144	newly 5 weeks cumul	56.	159.56	{49., 344.}	190.071	0.360424	newly 51 weeks cumul	56.	163.88	{51., 349.}	197.071	0.367634
25	newly 3 weeks spec	333.	907.115	{354., 1516.}	173.955	0.334696	newly 3 weeks cumul	1455.	3774.71	{1547., 6291.}	161.302	0.324887	newly 5 weeks spec	128.	481.981	{175., 756.}	276.547	0.458915	newly 5 weeks cumul	1803.	4923.19	{1991., 8129.}	174.508	0.341301	newly 42 weeks cumul	2085.	6163.23	{2420., 10217.}	196.471	0.369108
26	newly 3 weeks spec	134.	619.167	{268., 1096.}	362.065	0.563047	newly 3 weeks cumul	702.	2783.26	{1142., 4812.}	296.476	0.501261	newly 5 weeks spec	48.	296.148	{119., 522.}	516.975	0.680024	newly 5 weeks cumul	832.	3511.72	{1456., 6083.}	322.082	0.526437	newly 44 weeks cumul	1092.	4166.96	{1742., 7193.}	281.59	0.481345
27	newly 3 weeks spec	208.	907.115	{354., 1516.}	336.113	0.531663	newly 3 weeks cumul	1021.	3774.71	{1547., 6291.}	269.707	0.469835	newly 5 weeks spec	93.	481.981	{175., 756.}	418.259	0.597642	newly 5 weeks cumul	1280.	4923.19	{1991., 8129.}	284.624	0.483228	newly 42 weeks cumul	1520.	6163.23	{2420., 10217.}	305.476	0.502337
28	newly 3 weeks spec	419.	485.674	{142., 980.}	74.8127	0.287296	newly 3 weeks cumul	1620.	2188.21	{761., 4592.}	80.056	0.261381	newly 5 weeks spec	296.	229.279	{72., 490.}	66.5855	0.377031	newly 5 weeks cumul	2248.	2747.95	{909., 5825.}	75.5855	0.274806	newly 43 weeks cumul	3114.	3223.42	{1039., 6784.}	69.816	0.302195
29	newly 3 weeks spec	193.	192.024	{61., 366.}	70.6685	0.309762	newly 3 weeks cumul	755.	974.683	{316., 1887.}	77.1992	0.252499	newly 5 weeks spec	61.	79.2195	{20., 127.}	84.1663	0.29737	newly 5 weeks cumul	915.	1179.54	{404., 2223.}	77.8782	0.256114	newly 45 weeks cumul	1584.	1303.85	{434., 2388.}	66.6266	0.345414
30	newly 3 weeks spec	34.	409.966	{126., 693.}	1105.78	0.916606	newly 3 weeks cumul	205.	1990.53	{575., 3311.}	870.992	0.833199	newly 5 weeks spec	6.	171.259	{50., 307.}	2754.31	1.28831	newly 5 weeks cumul	220.	2430.66	{689., 4033.}	1004.84	0.887565	newly 47 weeks cumul	225.	2736.76	{800., 4531.}	1116.34	0.928092
31	newly 3 weeks spec	208.	546.255	{225., 925.}	166.264	0.321052	newly 3 weeks cumul	1042.	2436.51	{957., 4122.}	138.91	0.290568	newly 5 weeks spec	80.	257.255	{90., 429.}	225.346	0.393415	newly 5 weeks cumul	1231.	3063.04	{1194., 5046.}	153.188	0.30937	newly 44 weeks cumul	1394.	3605.38	{1373., 5847.}	162.693	0.320914
32	newly 3 weeks spec	553.	645.827	{271., 1362.}	56.0405	0.216119	newly 3 weeks cumul	1991.	2795.77	{1148., 5810.}	66.1206	0.211311	newly 5 weeks spec	288.	325.731	{143., 668.}	55.4888	0.221466	newly 5 weeks cumul	2687.	3582.52	{1476., 7407.}	62.7974	0.21119	newly 42 weeks cumul	3562.	4380.69	{1827., 9013.}	58.2829	0.213267
33	newly 3 weeks spec	0.1	5.75556	{0., 13.}	5677.78	1.43069	newly 3 weeks cumul	6.	54.6222	{14., 129.}	810.37	0.800791	newly 5 weeks spec	0.1	1.48889	{0., 6.}	1504.44	0.577581	newly 5 weeks cumul	6.	58.8889	{14., 138.}	881.481	0.829715	newly 52 weeks cumul	6.	60.5778	{14., 141.}	909.63	0.840133
34	newly 3 weeks spec	24.	88.1346	{29., 179.}	267.869	0.471503	newly 3 weeks cumul	66.	496.019	{207., 1021.}	651.544	0.793442	newly 5 weeks spec	13.	29.1923	{11., 49.}	132.249	0.307804	newly 5 weeks cumul	105.	577.846	{236., 1152.}	450.33	0.657616	newly 48 weeks cumul	110.	615.827	{249., 1218.}	459.843	0.665351
35	newly 3 weeks spec	0.1	6.95918	{1., 18.}	6863.27	1.63543	newly 3 weeks cumul	0.1	58.7347	{14., 122.}	58634.7	2.60863	newly 5 weeks spec	0.1	1.20408	{0., 5.}	1218.37	0.569771	newly 5 weeks cumul	0.1	62.9796	{15., 138.}	62879.6	2.6372	newly 52 weeks cumul	0.1	63.7755	{15., 142.}	63675.5	2.64212
36	newly 3 weeks spec	107.	12.5814	{2., 29.}	88.2417	1.14774	newly 3 weeks cumul	314.	104.93	{35., 231.}	67.9899	0.592599	newly 5 weeks spec	47.	2.97674	{0., 9.}	93.6665	1.72853	newly 5 weeks cumul	444.	114.186	{40., 250.}	74.2824	0.703636	newly 52 weeks cumul	542.	116.047	{40., 250.}	78.5892	0.784464
37	newly 3 weeks spec	66.	312.843	{104., 578.}	374.005	0.567688	newly 3 weeks cumul	452.	1485.06	{569., 2572.}	229.021	0.414246	newly 5 weeks spec	38.	133.902	{46., 192.}	252.786	0.435586	newly 5 weeks cumul	556.	1825.33	{683., 3108.}	228.756	0.412323	newly 45 weeks cumul	581.	2081.69	{766., 3513.}	258.294	0.448251
38	newly 3 weeks spec	0.1	27.2857	{5., 65.}	27185.7	2.27389	newly 3 weeks cumul	6.	218.51	{70., 497.}	3541.84	1.45234	newly 5 weeks spec	0.1	6.46939	{1., 15.}	6381.63	1.55625	newly 5 weeks cumul	6.	239.469	{77., 557.}	3891.16	1.49124	newly 52 weeks cumul	6.	245.857	{79., 578.}	3997.62	1.5025
39	newly 3 weeks spec	62.	34.2381	{6., 71.}	55.6068	0.456595	newly 3 weeks cumul	248.	227.024	{67., 462.}	62.932	0.32779	newly 5 weeks spec	8.	10.1905	{1., 22.}	86.9048	0.451078	newly 5 weeks cumul	278.	255.786	{72., 534.}	63.3008	0.330102	newly 49 weeks cumul	290.	266.786	{73., 560.}	63.5878	0.331502
40	newly 3 weeks spec	1.	32.5185	{8., 72.}	3151.85	1.27933	newly 3 weeks cumul	22.	229.481	{53., 442.}	943.098	0.817027	newly 5 weeks spec	0.1	8.14815	{0., 14.}	8070.37	1.55546	newly 5 weeks cumul	22.	253.778	{64., 487.}	1053.54	0.861963	newly 51 weeks cumul	22.	261.037	{64., 504.}	1086.53	0.873561
41	newly 3 weeks spec	9.	39.6296	{12., 65.}	344.444	0.510398	newly 3 weeks cumul	150.	281.852	{81., 522.}	110.247	0.254081	newly 5 weeks spec	0.1	8.42593	{1., 15.}	8337.04	1.64552	newly 5 weeks cumul	158.	309.352	{89., 561.}	117.382	0.265182	newly 52 weeks cumul	158.	318.685	{93., 576.}	122.023	0.269233
42	newly 3 weeks spec	40.	106.278	{38., 174.}	172.917	0.376463	newly 3 weeks cumul	178.	617.296	{222., 1026.}	247.503	0.459945	newly 5 weeks spec	2.	35.0556	{14., 69.}	1652.78	1.15356	newly 5 weeks cumul	187.	713.315	{256., 1213.}	281.868	0.500158	newly 49 weeks cumul	187.	758.685	{266., 1314.}	305.714	0.52623
43	newly 3 weeks spec	34.	53.1579	{11., 113.}	97.936	0.263373	newly 3 weeks cumul	177.	365.368	{107., 720.}	123.947	0.249595	newly 5 weeks spec	7.	13.7193	{3., 25.}	129.073	0.347911	newly 5 weeks cumul	204.	406.228	{123., 809.}	119.083	0.247975	newly 52 weeks cumul	209.	419.333	{124., 841.}	120.129	0.249038
44	newly 3 weeks spec	5.	27.96	{7., 68.}	462.4	0.621365	newly 3 weeks cumul	94.	194.62	{72., 398.}	119.34	0.263676	newly 5 weeks spec	0.1	7.42	{1., 16.}	7328.	1.63658	newly 5 weeks cumul	94.	216.2	{77., 465.}	138.723	0.287114	newly 51 weeks cumul	94.	222.62	{77., 485.}	144.787	0.295664
45	newly 3 weeks spec	93.	199.58	{61., 390.}	127.462	0.294551	newly 3 weeks cumul	558.	1043.34	{322., 1894.}	103.796	0.262932	newly 5 weeks spec	65.	76.1	{25., 144.}	60.2769	0.232141	newly 5 weeks cumul	698.	1242.7	{391., 2296.}	96.6447	0.253751	newly 47 weeks cumul	726.	1362.86	{434., 2536.}	104.416	0.263924
46	newly 3 weeks spec	37.	486.159	{182., 993.}	1213.94	0.992907	newly 3 weeks cumul	384.	2364.98	{990., 4767.}	515.881	0.670999	newly 5 weeks spec	4.	198.46	{72., 383.}	4861.51	1.56531	newly 5 weeks cumul	404.	2873.56	{1209., 5795.}	611.276	0.73182	newly 48 weeks cumul	409.	3233.7	{1337., 6611.}	690.635	0.776833
47	newly 3 weeks spec	33.	237.842	{84., 421.}	620.734	0.727062	newly 3 weeks cumul	146.	1256.37	{449., 2098.}	760.526	0.813133	newly 5 weeks spec	17.	87.614	{31., 163.}	415.377	0.587021	newly 5 weeks cumul	191.	1488.12	{540., 2498.}	679.122	0.769896	newly 48 weeks cumul	201.	1628.26	{601., 2736.}	710.081	0.78641
48	newly 3 weeks spec	101.	809.649	{313., 1455.}	701.633	0.824422	newly 3 weeks cumul	455.	3507.33	{1363., 6201.}	670.842	0.812135	newly 5 weeks spec	16.	411.263	{147., 744.}	2470.39	1.32764	newly 5 weeks cumul	527.	4495.7	{1752., 7898.}	753.074	0.85539	newly 43 weeks cumul	536.	5485.68	{2103., 9524.}	923.449	0.934388
49	newly 3 weeks spec	21.	52.6977	{15., 86.}	166.667	0.316507	newly 3 weeks cumul	128.	346.512	{114., 546.}	178.016	0.303661	newly 5 weeks spec	0.1	15.5581	{3., 31.}	15462.8	1.95126	newly 5 weeks cumul	131.	391.326	{129., 615.}	203.586	0.33259	newly 49 weeks cumul	131.	408.953	{134., 649.}	216.652	0.349151
50	newly 3 weeks spec	8.	43.2558	{9., 130.}	445.349	0.544775	newly 3 weeks cumul	96.	290.465	{76., 873.}	212.597	0.348263	newly 5 weeks spec	0.1	12.6512	{1., 39.}	12560.5	1.7578	newly 5 weeks cumul	102.	326.93	{86., 989.}	229.503	0.363828	newly 50 weeks cumul	102.	340.488	{86., 1030.}	241.974	0.375821
